# Extracellular volume fraction measurements derived from the longitudinal relaxation of blood-based synthetic hematocrit may lead to clinical errors in 3 T cardiovascular magnetic resonance

**DOI:** 10.1186/s12968-018-0475-6

**Published:** 2018-08-09

**Authors:** Yongning Shang, Xiaochun Zhang, Xiaoyue Zhou, Jian Wang

**Affiliations:** 1 Department of Radiology, Southwest Hospital, Third Military Medical University, Gaotanyan Street No. 30, Shapingba district, Chongqing, China; 2 MR Collaboration, Siemens Healthcare Ltd., Shanghai, China

**Keywords:** 3 T cardiovascular magnetic resonance, T1 mapping, Synthetic hematocrit, Extracellular volume fraction

## Abstract

**Background:**

The extracellular volume (ECV), derived from cardiovascular magnetic resonance (CMR) T1 mapping, is a biomarker of the extracellular space in the myocardium. The hematocrit (HCT), measured from venipuncture, is required for ECV measurement. We test the clinic values of synthetic ECV, which is derived from the longitudinal relaxation of blood–based (T1_blood_) synthetic hematocrit in 3 T CMR.

**Methods:**

A total of 226 subjects with CMR T1 mapping and HCT measurement taken on the same day as the CMR were retrospectively enrolled and randomly split into derivation (*n* = 121) and validation (*n* = 105) groups, comprising healthy subjects (*n* = 45), type 2 diabetes mellitus (T2DM) patients (*n* = 60), hypertrophic cardiomyopathy (HCM) patients (*n* = 93), and 28 other patients. Correlation of T1_blood_ with the measured HCT (HCTm) was established in the derivation group and used in both the derivation and the validation groups. The relationships between the ECV values derived from both the synthetic HCT (HCTsyn) and HCTm were explored. In addition, the differences in the ECV values among the HC, T2DMs, and HCMs were compared.

**Results:**

Regression between the HCTm and 1/T1_blood_ was linear (R^2^ = 0.19, *p* < 0.001), and the regression equation was: HCTsyn = [561.6*(1/T1blood)] + 0.098 in the derivation group. The measured ECV (ECVm) was strongly correlated with the synthetic ECV (ECVsyn) (R^2^ = 0.87, *p* < 0.001) and mildly correlated with the difference between the ECVsyn and ECVm (R^2^ = 0.10, *p* < 0.001) in the derivation group. Also in this group, the ECVm was larger in T2DMs than that in healthy cohort (29.1 ± 3.1% vs. 26.4 ± 2.4%, *p* = 0.002), whereas, the ECVsyn did not differ between T2DMs and healthy cohort (28.3 ± 2.9% vs. 26.9 ± 2.2%, *p* = 0.064). Compared with the healthy cohort, the HCMs were associated with higher ECVsyn and ECVm of the mid-ventricle in both the derivation and the validation groups. Using our center’s normal cut-off of 31.8%, the use of ECVsyn would lead to a 6–25% incorrect categorization of patients in the derivation and validation groups.

**Conclusions:**

ECVsyn derived from HCTsyn may lead to clinical errors in 3 T CMR, especially for patients who have only a subtle elevation in ECV.

**Electronic supplementary material:**

The online version of this article (10.1186/s12968-018-0475-6) contains supplementary material, which is available to authorized users.

## Background

Cardiovascular magnetic resonance (CMR) T1 mapping allows for the quantitative measurement of myocardial longitudinal relaxation time (T1) [1]. The extracellular volume fraction (ECV), which is derived from native and post-contrast T1 mapping, reflects the size of the extracellular space in the myocardium. ECV strongly correlates with the histological measurements of the extracellular matrix [2] and can be used as an important diagnostic biomarker of disease [3], as well as for observation of disease progression [4] and prognosis [5, 6]. T1 mapping, together with the ECV, introduced a new frontier in radiology and cardiology, being independent of cardiac function and enabling the quantification of important tissue properties of both the local and global myocardium [7].

Hematocrit (HCT) measurement is necessary when calculating the ECV. However, the additional blood test is cumbersome and costly, and it delays the process of CMR examination and ECV calculation. In addition, it may cause difficulties for retrospective studies, particularly for those patients without a measured HCT.Treibel et al. [8] reported a linear correlation between the HCT and 1/T1_blood_ at 1.5 T CMR. This correlation can be applied to estimate the synthetic HCT (HCTsyn) and subsequently, the ECV. The study results indicated a very strong correlation between synthetic ECV (ECVsyn) and measured ECV (ECVm), which is consistent with another study at both 1.5 T and 3 T CMR [9]. Therefore, they concluded that HCTsyn can lead to an accurate ECV without the need for blood sampling. However, Raucci et al. [10] used the same method to study pediatric and young adult patients at 1.5 T CMR. Their results showed that HCTsyn may cause clinically significant errors in ECV measurement. Thus, whether it can be used in clinical routine workflow or not is still a subject of dispute, especially at 3 T CMR.

The goals of the present study were to explore whether T1 of blood-based (T1_blood_) HCTsyn and ECVsyn can be applied in 3 T CMR and to assess the feasibility of its clinical validation.

## Methods

### Study patients

This retrospective study was approved by the Institutional Review Board of our hospital, and all subjects provided written informed consent. The patients and healthy subjects who underwent CMR native and post-contrast T1 mapping with the modified Look-Locker inversion recovery (MOLLI) sequence and an HCT measurement within the same day of CMR were enrolled. A total of 226 subjects were eligible. They were randomly split into derivation (*n* = 121) and validation groups (*n* = 105). Healthy subjects (*n* = 45) had normal systolic (< 140 mmHg) and diastolic (< 90 mmHg) blood pressure, normal electrocardiogram (ECG) and CMR results, and no history of cardiovascular disease. Type 2 diabetes mellitus (T2DM) patients (*n* = 60) were diagnosed according to World Health Organization criteria [11], and hypertrophic cardiomyopathy (HCM) patients (*n* = 93) met the European Society of Cardiology criteria [12]. Twenty eight other patents were enrolled, including hypertension (*n* = 4), cardiac amyloidosis (*n* = 2), chronic myocardial infarction (*n* = 6), left ventricular noncompaction (LVNC, *n* = 1), dilated cardiomyopathy (DCM, n = 9), arrhythmogenic right ventricular cardiomyopathy (ARVC, *n* = 2) and myocarditis (*n* = 4).

### HCT measurements

Whole blood was drawn in all subjects by venipuncture and HCT was analyzed using a Sysmex XN-1000 hematology analyzer (Sysmex Corporation, Kobe, Japan) [13].

### Cardiovascular magnetic resonance protocols

CMR was performed on a 3 T MAGNETOM Trio MR scanner (Siemens Healthineers, Erlangen, Germany) with a 6-channel body arrayed coil plus a 6-channel spine arrayed coil. A prototype target shimming method for patient-specific, localized shimming in the heart was used to improve field uniformity.

Short-axis cine images covering the entire left ventricle (LV) were performed using an electrographic-gated, breath-hold, balanced steady-state free-precession (bSSFP) sequence. Segmented late gadolinium enhancement (LGE) images covering the entire LV were performed using the Phase Sensitive Inversion Recovery (PSIR) sequence approximately 10 min after a bolus administration of 0.2 mmol/kg gadoteric acid meglumine bolus (Dotarem, Guerbet, BP7400, F95943, Roissy CdG Cedex, France).

A breath-hold, ECG-gated, MOLLI prototype sequence with a 5b (3b) 3b and 4b (1b) 3b (1b) 2b sampling pattern was performed for native and post-contrast T1 mapping, respectively, with a bSSFP readout, FOV 400 × 300 mm^2^, matrix 256 × 166, TR/TE 301.7/1.09 ms, acquisition window duration 302 ms (native), 382 ms (post-contrast), flip angle 35 degrees and 6 mm thickness. Basal, mid-ventricular, and apical LV short-axis images were acquired before and approximately 15 min after the administration of contrast. T1 maps were generated online from the MOLLI images after the motion correction (MOCO).

### Synthetic and measured ECV

All the cine and T1 maps were transferred to the cvi42 software (Circle Cardiovascular Imaging Inc., Calgary, Alberta, Canada) for offline analysis. The LV endo- and epi-myocardial borders on the T1 maps were manually delineated with attention being paid to avoiding partial-volume effects from the blood pool and epicardial fat. The regions of interest (ROIs) were manually drawn with care in the LV cavity to avoid the papillary muscles and myocardium. Native and post-contrast T1 values of the blood sample and 16 myocardial segments were obtained. Sixteen-segmental myocardial ECV values were calculated from native and post-contrast T1 maps using the following formula [6]:

$$ ECV=\left(1-\mathrm{HCT}\right)\frac{\frac{1}{\mathrm{T}1\ \mathrm{myo}\ \mathrm{post}}-\frac{1}{\mathrm{T}1\ \mathrm{myo}\ \mathrm{native}}\ }{\frac{1}{\mathrm{T}1\ \mathrm{blood}\ \mathrm{post}}-\frac{1}{\mathrm{T}1\ \mathrm{blood}\ \mathrm{native}}} $$

According to Treibel et al., there is a linear relationship between the longitudinal relativity (R1 = 1/T1) of blood and blood HCT [8]. The linear equation between blood R1 and HCTm was derived in the derivation group and used to estimate a HCTsyn and calculate an ECVsyn (Additional file 1: Figure S7). Additionally, the published equation between blood R1 and HCTm in 3 T CMR [9] was also used to estimate a HCTsyn and calculate an ECVsyn. The HCTsyn and ECVsyn were subsequently compared separately with HCTm and ECVm.

### Statistical analysis

Categorical data were presented as percentages. The Kolmogorov-Smirnov test was used to test the normality of the variables. Data that did not fit normality were summarized as median (interquartile range). Continuous and normal variables were presented as mean and standard deviation (SD). The robustness of the T1_blood_-based HCTsyn calculation was evaluated using the bootstrap trials: a subset of the samples (90%) was randomly resampled 100 times, and R^2^ was performed for each subset [14]. The differences between the means were compared using the unpaired t-test, paired t-test, and Bland-Altman method. The relationships between bivariates were analyzed using Pearson’s method. Intraclass correlation coefficients were used to determine how strongly ECVsyn and ECVm of the 16-segment myocardium correlated with each other in the derivation and validation groups. The statistical tests were two-tailed, and statistical significance was defined as *P* < 0.05. Data were analyzed using SPSS (version 21.0, Statistical Package for the Social Sciences, International Business Machines, Inc., Armonk, New York, USA) and GraphPad Prism (version 6.01, GraphPad Software, Inc., La Jolla, California, USA).

## Results

### Participant characteristics

Demographic and clinical data are summarized in Table 1. There were equivalent age, gender, LV structure, function, HCT (derivation group, 40.4 ± 4.7%, range 25.7 to 54.6% vs. validation group, 40.9 ± 5.1%, range 25.4 to 52.2%, *p* = 0.386) and ECV (derivation group, 30.3 ± 6.3% vs. validation group, 29.0 ± 4.2%, *p* = 0.059) values between the derivation and validation groups.

**Table 1:** Patient characteristics for Derivation and Validation groups

	Derivation (*n* = 121)	Validation (*n* = 105)	*P* value
Age, years	49.8 ± 12.4	50.8 ± 13.0	0.554
Male, *n* (%)	66 (54.6)	65 (61.9)	0.264
BSA, m^2^	1.70 ± 0.22	1.72 ± 0.17	0.266
Healthy volunteer	18	27	
Type 2 diabetes mellitus	34	26	
Hypertrophic cardiomyopathy	49	44	
Others	20	8	
EDVi, ml/m^2^	71.0 ± 26.3	71.1 ± 25.6	0.968
ESVi, ml/m^2^	32.5 ± 26.5	30.8 ± 25.7	0.629
SVi, ml/m^2^	38.5 ± 8.8	40.3 ± 8.5	0.120
EF, %	56.9 ± 11.3	58.9 ± 10.0	0.174
CI, L/min/m^2^	2.82 ± 0.77	2.95 ± 0.62	0.159
LVMi, g/m^2^	80.8 ± 40.6	77.4 ± 34.3	0.506
Hematocrit, %	40.4 ± 4.7	40.9 ± 5.1	0.386
Myocardial native T1, ms	1285 ± 859	12,657 ± 75	0.055
Myocardial post-contrast T1, ms	504 ± 78	514 ± 54	0.273
Blood native T1, ms	1846 ± 120	1827 ± 130	0.242
Blood post-contrast T1, ms	343 ± 78	345 ± 54	0.883
ECV, %	30.3 ± 6.3	29.0 ± 4.2	0.059

*BSA* body surface area, *EDVi* end-diastolic volume index, *ESVi* end-systolic volume index, *SVi* stoke volume index, *EF* ejection fraction, *CI* cardiac index, *LVMi* left ventricular mass index, *ECV* extracellular volume

### Relationship between HCT and blood T1

**Fig. 1:**
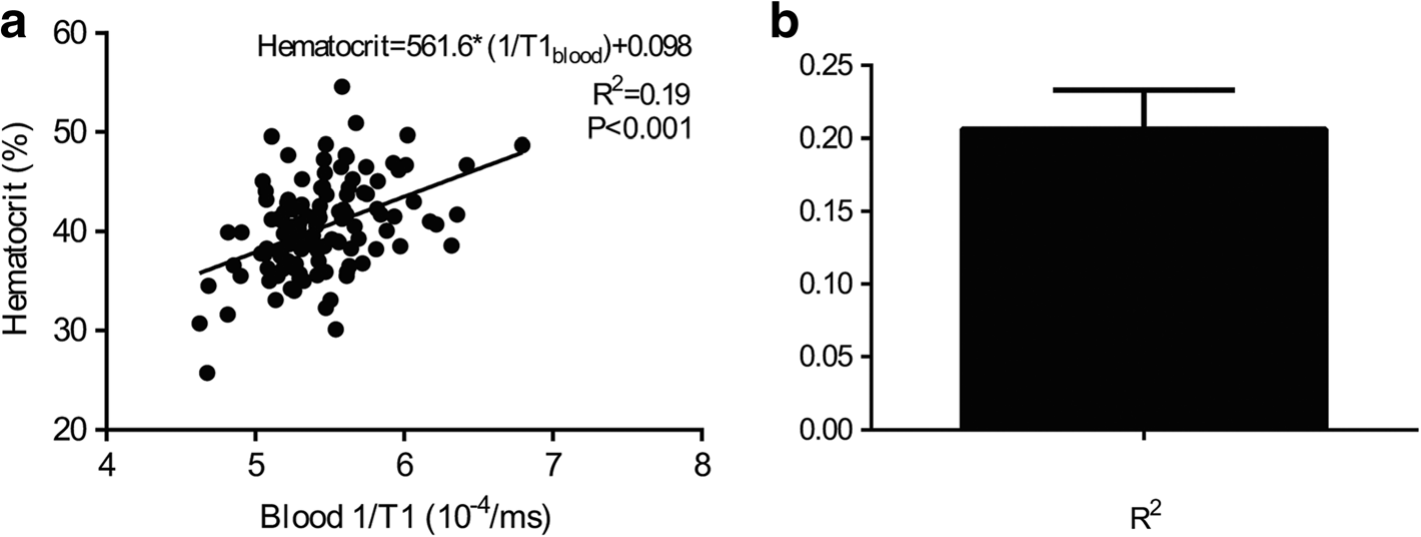
Correlation between blood 1/T1 and hematocrit (HCT) and robustness of R^2^ in the derivation group. Left panel showing the linear regression line between the HCT and 1/T1_blood native_ with R^2^ (p and regression equations shown in the graph). Right panel shows the R^2^ distribution based on 100-times bootstrap correlation analysis

The regression between the HCT and R1_blood_ (1/T1_blood_) was linear (R^2^ = 0.19, *p* < 0.001), and the regression equation was HCTsyn = [562*(1/T1_blood_)] + 0.098 (Fig. 1a) in the derivation group. Bootstrap correlation analysis showed that the average value of R^2^ of T1_blood_-based HCTsyn calculation was 0.21 ± 0.03 (range, 0.12 to 0.27, Fig. 1b).

### Correlation between HCTm and HCTsyn, and ECVm and ECVsyn in the derivation and validation groups

**Fig. 2:**
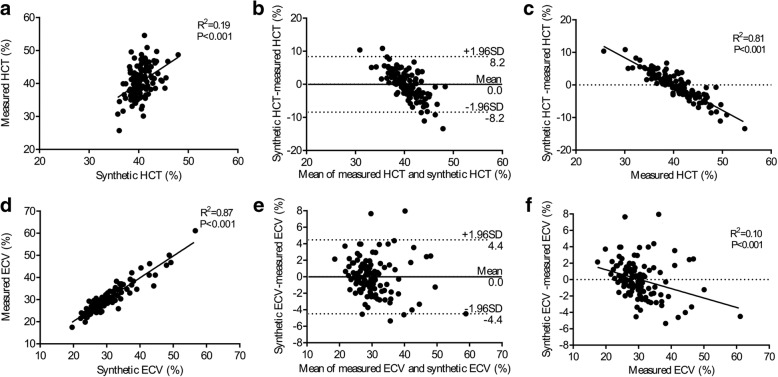
Correlation among the measured hematocrit (HCTm), synthetic hematocrit (HCTsyn), measured extracellular volume fraction (ECVm), synthetic extracellular volume (ECVsyn) in the derivation group. There was modest correlation between the HCTsyn and HCTm (**a**) and strong correlation between the ECVsyn and ECVm (**d**). Bland-Altman analysis indicated minimal bias between the HCTsyn and HCTm (**b**) and between the ECVsyn and ECVm (**e**). HCTm strongly correlated with (HCTsyn –HCTm) (**c**) and the ECVm poorly correlated with (ECVsyn – ECVm) (**f**). HCTsyn was not associated with (HCTsyn –HCTm) in the derivation group (R^2^ < 0.01, *p* > 0.999) and validation group (R^2^ < 0.01, *p* = 0.731)

**Fig. 3:**
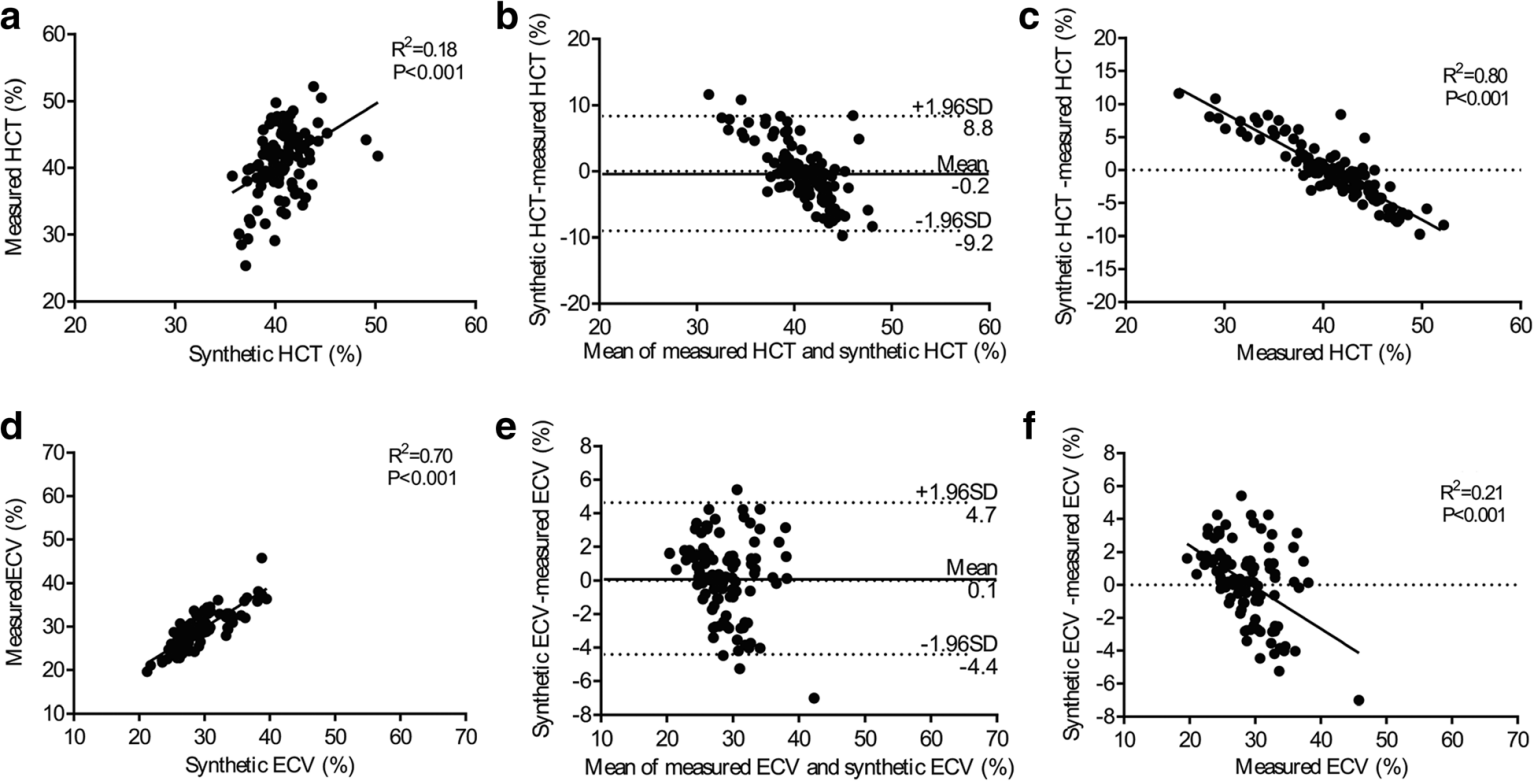
Correlation among the HCTm, HCTsyn, ECVm, ECVsyn in the validation group. There was modest correlation between the HCTsyn and HCTm (**a**) and strong correlation between the ECVsyn and ECVm (**d**). Bland-Altman analysis indicated minimal bias between the HCTsyn and HCTm (**b**) and between the ECVsyn and ECVm (**e**). HCTm strongly correlated with (HCTsyn –HCTm) (**c**) and the ECVm modestly correlated with (ECVsyn – ECVm) (**f**)

Using the equation for the calculation of HCTsyn, there was modest correlation between HCTsyn and HCTm in the derivation group (Slope = 1.00, R^2^ = 0.19, *p* < 0.001, Fig. 2a) and slightly weaker correlation in the validation group (Slope = 0.93, R^2^ = 0.18, *p* < 0.001, Fig. 3a). Bland-Altman analysis indicated 0.0% bias (− 8.2 to 8.2%, Fig. 2b) in the derivation group and − 0.2% bias (− 9.2 to 8.8%, Fig. 3b) in the validation group. Interestingly, the difference between HCTsyn and HCTm strongly correlated with HCTm in both the derivation group (Slope = − 0.8082, R^2^ = 0.81, *p* < 0.001, Fig. 2c) and validation group (Slope = − 0.8102, R^2^ = 0.80, *p* < 0.001, Fig. 3c). But, the difference between HCTsyn and HCTm was not associated with HCTsyn in the derivation group (R^2^ < 0.01, *p* > 0.999) and validation group (R^2^ < 0.01, *p* = 0.731).

Regarding ECVsyn and ECVm, there was a strong correlation between ECVsyn and ECVm in the derivation group (Slope = 0.98, R^2^ = 0.87, *p* < 0.001, Fig. 2d) and slightly weaker correlation in the validation group (Slope = 0.94, R^2^ = 0.70, *p* < 0.001, Fig. 3d). Bland-Altman analysis indicated 0.0% bias (− 4.4 to 4.4%, Fig. 2e) in the derivation group and 0.1% bias (− 4.4 to 4.7%, Fig. 3e) in the validation group. The difference between ECVsyn and ECVm poorly correlated with ECVm in the derivation group (Slope = − 0.11, R^2^ = 0.10, *p* < 0.001, Fig. 2f) and moderately correlated with ECVm in the validation group (Slope = − 0.25, R^2^ = 0.21, *p* < 0.001, Fig. 3f). The mean difference and intraclass correlation coefficients (ICCs) for the 16-segment myocardial extracellular volume fraction are summarized in Additional file 2: Table S6.

**Fig. 4:**
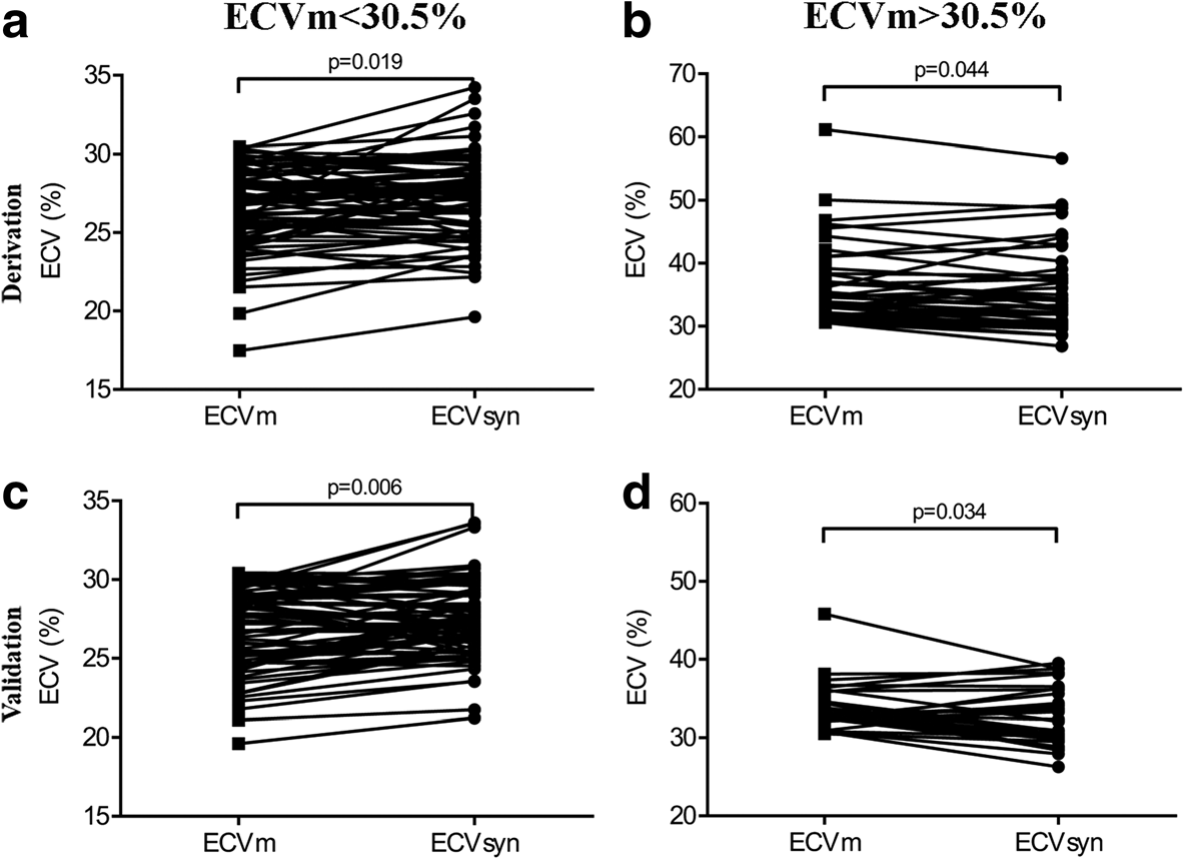
Comparison between the ECVm and ECVsyn in groups with the ECVm < 30.5 and > 30.5%. In the group with the ECVm < 30.5%, the paired t-test demonstrated that the ECVsyn was larger than the ECVm in the derivation group (**a**) and validation group (**c**). In the group with the ECV > 30.5%, the paired t-test demonstrated that the ECVsyn was smaller than the ECVm in the derivation group (**b**) and validation group (**d**)

According to the regression equation between ECVm and the difference between ECVsyn and ECVm in the derivation group ((ECVsyn-ECVm) = − 0.11*ECvm + 0.035, Fig. 2f), when ECVm = 30.5%, the difference = 0, suggesting that when ECVm is smaller than 30.5%, ECVsyn is larger than ECVm, and vice versa. All the participants were subsequently divided into two groups, with a cut-off of 30.5% in both the derivation and validation groups. In participants with ECV < 30.5%, the paired t-test demonstrated that ECVsyn was larger than ECVm in both the derivation group (*p* = 0.019, Fig. 4a) and the validation group (*p* = 0.006, Fig. 4c). In participants with ECV > 30.5%, the paired t-test demonstrated that ECVsyn was smaller than ECVm in the derivation group (*p* = 0.044, Fig. 4b) and validation group (*p* = 0.034, Fig. 4d).

The results of the published model were almost equivalent with the results of the local model and presented in Additional file 3: Figure S1, Additional file 4: Figure S2 and Additional file 5: Figure S3.

### Comparison of ECVsyn and ECVm among healthy subjects, T2DMs, and HCMs

**Fig. 5:**
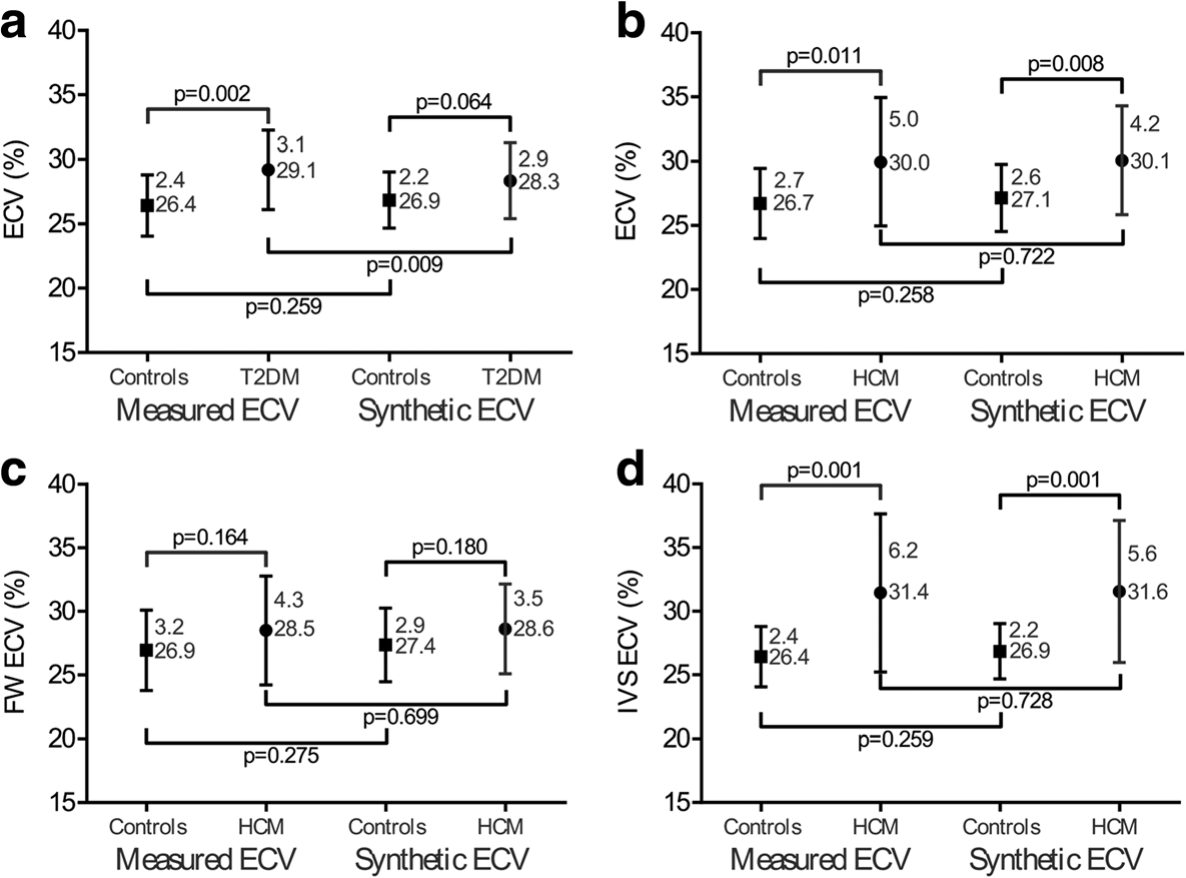
Comparison of the ECVsyn and ECVm among the healthy subjects and patients in the derivation group. The ECVm was larger in T2DMs patients than healthy subjects (**a**); however, the ECVsyn did not differ (**a**). Compared with the healthy subjects, the HCM patients had a higher ECVsyn and ECVm of the mid-ventricle (**b**) and interventricular septum (**d**). The ECVm and ECVsyn of the free wall in the HCM patients did not differ with those in the healthy subjects (**c**)

**Fig. 6:**
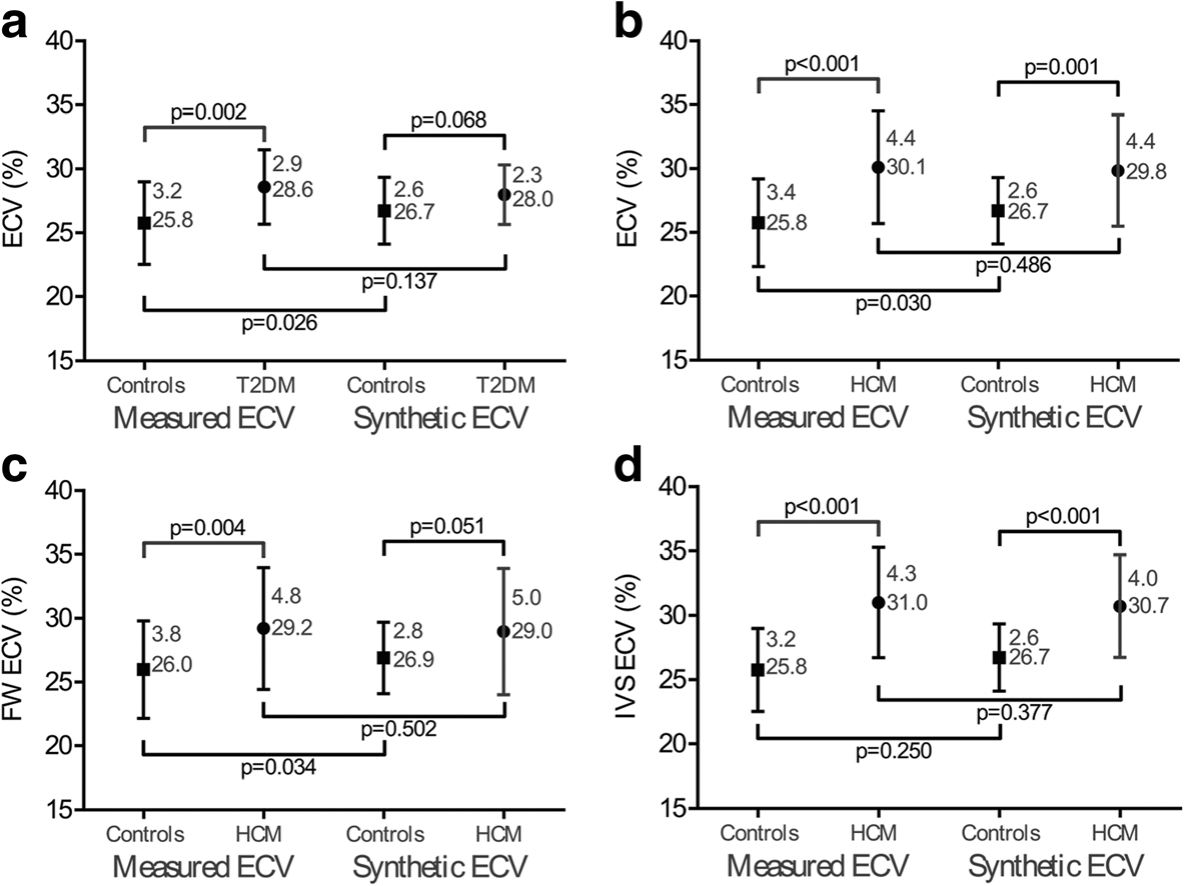
Comparison of the ECVsyn and ECVm among the healthy subjects and patients in the validation group. The ECVm was larger in T2DMs patients than healthy subjects (**a**); however, the ECVsyn did not differ (**a**). Compared with the healthy subjects, the HCM patients had a higher ECVsyn and ECVm of the mid-ventricle (**b**) and interventricular septum (**d**). The ECVm of the free wall in the HCM patients was larger than in the healthy subjects, but the ECVsyn was equivalent (**c**)

The ECVm of the interventricular septum was larger in T2DMs than healthy subjects in the derivation group (T2DM, 29.1 ± 3.1% vs. healthy subjects, 26.4 ± 2.4%, *p* = 0.002, Fig. 5a) and in the validation group (T2DM, 28.6 ± 2.9% vs. healthy subjects 25.8 ± 3.2%, *p* = 0.002, Fig. 6a). However, the ECVsyn did not differ between T2DMs and healthy subjects in the derivation group (T2DM, 28.3 ± 2.9% vs. healthy subjects, 26.9 ± 2.2%, *p* = 0.064, Fig. 5a) and the validation group (T2DM, 28.0 ± 2.3% vs. healthy subjects, 26.7 ± 2.6%, *p* = 0.068, Fig. 6a).

For the HCM patients, the ECVsyn and ECVm of the mid-ventricle, interventricular septum, and free wall were separately obtained and compared. In comparison with the healthy subjects, the HCM patients had remarkably higher ECVsyn and ECVm values for the mid-ventricle and interventricular septum in both the derivation and validation groups (Figs. 5b, d and 6b, d). In the derivation group, both the ECVm and ECVsyn of the free wall in the HCM patients did not differ with those in the healthy subjects (Fig. 5c). In the validation group, the ECVm of the free wall was larger in the HCM patients than in the healthy subjects, whereas the ECVsyn did not differ in the HCM patients and the healthy subjects (Fig. 6c).

The results for published model were almost equivalent with those for the local model and presented in Additional file 6: Figure S4 and Additional file 7: Figure S5.

### Use of ECVm and ECVsyn to categorize patients with abnormal ECV

The ECV results of the T2DM and HCM patients were separately compared to our laboratory normal cut-off of 31.8%. This cut-off value was set at 2 standard deviation above the mean ECVm derived from all 45 healthy subjects (mean, 26.0%, and SD, 2.9%). In the derivation group, the number (percentage) that had an ECVm larger than 31.8% was 13 (38%) of the T2DM patients, 19 (39%) of the HCM (mid-ventricle) patients, 24 (49%) of the HCM (interventricular septum) patients, and 15(31%) of the HCM (free wall) patients. For local model, the use of the ECVsyn led to a significantly incorrect categorization of patients, with a total miscategorized fraction ranging from 6 to 12% in the derivation group. The total miscategorized fraction was larger in the validation group, ranging from 12 to 25% (Table 2). For published model, the use of the ECVsyn led to a total miscategorized fraction ranging from 10 to 15% in the derivation group and 8 to 18% in the validation group (Table 3).

**Table 2:** Miscategorization of patients with abnormal ECV for local model

	False negative		False Positive		Total miscategorizations	
	derivation	validation	derivation	validation	derivation	validation
Healthy, *n* (%)	0 (0)	1 (4)	0 (0)	1 (4)	0 (0)	2 (7)
T2DM-ECVsyn, *n* (%)	2 (6)	3 (12)	1 (3)	0 (0)	3 (9)	3 (12)
HCM-ECVsyn-mid ventricle, *n* (%)	1 (2)	6 (14)	2 (4)	5 (11)	3 (6)	11 (25)
HCM-ECVsyn-IVS, *n* (%)	2 (4)	6 (14)	5 (10)	1 (2)	7 (9)	7 (16)
HCM-ECVsyn-FW, *n* (%)	3 (6)	3 (7)	3 (6)	3 (7)	6 (12)	6 (14)

*ECVsyn* synthetic ECV, *IVS* interventricular septum, *FW* free wall

**Table 3:** Miscategorization of patients with abnormal ECV for published model

	False negative		False Positive		Total miscategorizations	
	derivation	validation	derivation	validation	derivation	validation
Healthy, *n* (%)	0 (0)	1 (4)	0 (0)	1 (4)	0 (0)	2 (7)
T2DM-ECVsyn, *n* (%)	3 (9)	2 (8)	2 (6)	0 (0)	5 (15)	2 (8)
HCM-ECVsyn-mid ventricle, *n* (%)	3 (6)	4 (9)	2 (4)	4 (9)	5 (10)	8 (18)
HCM-ECVsyn-IVS, *n* (%)	2 (4)	6 (14)	4 (8)	1 (2)	6 (12)	7 (16)
HCM-ECVsyn-FW, *n* (%)	4 (8)	2 (5)	3 (6)	4 (9)	7 (14)	6 (14)

*ECVsyn* synthetic ECV, *IVS* interventricular septum, *FW* free wall

## Discussion

CMR ECV, which enables the quantification of the extracellular matrix in vivo, is being embraced as a useful imaging biomarker of diffuse fibrosis [15, 16]. HCT measurements, obtained from blood sampling, are essential but are burdensome for ECV quantification. Although great efforts have been made to explore the relationship between T1_blood_-based HCTsyn and ECVsyn, feasibility of performance and the clinical values of HCTsyn and ECVsyn is still a dispute at 3 T CMR. We assessed the equation of T1_blood_-based HCTsyn in a derivation population and subsequently applied it in a validation population.

The ECVsyn was strongly correlated with the ECVm, and the differences were minimal in both groups. However, the ECVsyn may erroneously eliminate the differences between T2DM patients and healthy subjects and between HCM patients and the healthy subjects in the free wall of myocardium. Moreover, the ECVsyn may miscategorize patients with an abnormal ECV, and the total miscategorized fraction was larger in the validation group than in the derivation group.

Our data showed a significant correlation between the HCT and 1/T1_blood_ at 3 T CMR, consistent with previous studies [17, 18]. According to the derived equation from the derivation group, the HCTsyn was modestly correlated with the HCTm in the validation group, verifying its applicability. With the condition of 1.5 T CMR, the correlation noted in Treibel’s study [8] and Fent’s study [9] was strong but was modest in Raucci’s study [10]. At 3 T CMR, Fent et al. [9] studied 218 patients (HCT, range 31 to 54%, comprising of 159 (73%) patients with rheumatoid arthritis, 33 (15%) patients with HCM and 26 (12%) healthy subjects) and the correlation was strong (R^2^ = 0.46), which is larger than our result. In the present study, 226 subjects (HCT, range 25.4 to 54.6%, including 45 (20%) healthy subjects, 60 (27%) patients with T2DM, 93 (41) patients with HCM and other 28 (12%) patients with different diseases) were enrolled and the correlation was modest. There are differences in spectrum of diseases and range of HCT between their and our study, which may lead to the discrepancy in the strength of the correlation. So, it is necessary to explore their relationship in a large, multi-center cohort study with diverse diseases and larger range of HCT.

Despite the modest correlation between the HCTsyn and HCTm, the ECVsyn was strongly correlated with the ECVm in both the derivation and validation groups. This trend found in our 3 T data was the same as that verified with 1.5 T [8–10]. Although the correlations between the ECVsyn and ECVm were strong overall, our values and those of Raucci were slightly weaker than those of Treibel and Fent.

ECV measurements were determined by five factors, including HCT, native and post-contrast T1 of the myocardium and blood. Regarding calculation of ECVm and ECVsyn, except for HCT, the other four factors remained unchanged. In other words, partition coefficient (λ = (1/T1_myo-post_ - 1/T1_myo-native_)/(1/T1_blood-post_ - 1/T1_blood-native_)) remained constant. Thus, although the correlation between HCTm and HCTsyn was relatively small, the correlation between ECVm and ECVsyn was relatively large, as the effect is alleviated by the unchanged λ in the equation [10].

Interestingly, we found that there was a weakly negative correlation between the ECVm and (ECVsyn - ECVm), with a cut-off value of ECVm = 30.5% in the derivation group. This suggested that when the ECVm is higher than 30.5%, the ECVsyn is lower than the ECVm. This condition was more pronounced in the validation group. We should pay much more attention when applying HCTsyn and ECVsyn to the other cohort. It is particularly important to note that because the ECV values in most of the diseases that affect the myocardium would become higher, such a condition may lead the ECVsyn to narrow the differences between the abnormal subjects and the healthy subjects.

While some conditions such as myocardial infarction, cardiac amyloid, and HCM with LGE show a significant increase in the ECV [19, 20], more subtle differences are seen in conditions with less myocardial damage, such as T2DM or HCM without LGE. Wong’s study [6] and our previous study [21] demonstrated that T2DM patients had a slightly but significant increased ECV. In the present study, there were minor but significant differences in the ECVm between the T2DM patients and the healthy subjects; however, the ECVsyn did not differ. This condition is equivalent for the ECV value in the free wall of the heart in the HCM patients in validation group. Regarding the ECV value in the mid-ventricle and interventricular septum of the HCM patients, there were major and significant differences in the ECVm and the differences in the ECVsyn remained significant. Therefore, when researchers study patients with a subtly elevated ECV, using the ECVsyn could lead to incorrect conclusions being made, which considerably limits its application for research. It would be prudent for researchers using the ECVsyn in patients with subtle elevated ECV value to note these precautions.

As for individual patients, using the ECVsyn could lead to significant miscategorization. The total miscategorized fraction ranged from 6 to 25% for local model and 8 to 18% for the published model in the derivation and validation group. It indicated that using the ECVsyn may cause incorrect treatment of many patients and a delay in the diagnosis and treatment of other patients. This also substantially limits the clinical utility of the ECVsyn.

### Limitations

There are limitations in our current study. First, although we tested our results separately in derivation and validation groups, the project was not a multicenter follow-up study. Second, the spectrum of diseases assessed in this study was not broad. T2DM and HCM patients were primarily recruited for frequent CMR in our center, and the number of diseases, such as myocardial infarction, cardiac amyloidosis and DCM, was relatively small. Third, this was a retrospective study, and the variability of the HCT measurements could therefore not be evaluated [8]. Four, although all subjects were randomly split into derivation and validation groups, the number of subjects in the validation group (*n* = 105) was relatively smaller than in the derivation group (*n* = 121) and the range of HCTm in the validation group (25.4 to 52.2%) was relatively narrower than in the derivation group (25.7 to 54.6%).

## Conclusions

T1_blood_-based synthetic HCT and ECV are useful for the assessment of health and disease in 3 T CMR. They may serve as a convenient and concise research tool, particularly in patients whose HCT measurements are not readily available. However, T1_blood_-based synthetic HCT and ECV may lead to incorrect conclusions for populations with a subtly elevated ECV in clinical research, such as in T2DM, and can lead to significant miscategorization for individual patients in clinical care.

## Additional files

Additional file 1: **Figure S7.** Diagram of synthetic HCT and ECV analysis of the left ventricular myocardium in a participant. (TIF 5299 kb)

Additional file 2: **Table S6.** Mean difference and intraclass correlation coefficients (ICCs) for 16-segment extracellular volume fraction. (DOCX 13 kb)

Additional file 3: **Figure S1.** Correlation among the HCTm, HCTsyn, ECVm, ECVsyn in the derivation group for published model. There was modest correlation between the HCTsyn and HCTm (A) and strong correlation between the ECVsyn and ECVm (D). Bland-Altman analysis indicated minimal bias between the HCTsyn and HCTm (B) and between the ECVsyn and ECVm (E). HCTm strongly correlated with (HCTsyn –HCTm) (C) and the ECVm poorly correlated with (ECVsyn – ECVm) (F). (TIF 8826 kb)

Additional file 4: **Figure S2.** Correlation among the HCTm, HCTsyn, ECVm, ECVsyn in the validation group for published model. There was modest correlation between the HCTsyn and HCTm (A) and strong correlation between the ECVsyn and ECVm (D). Bland-Altman analysis indicated minimal bias between the HCTsyn and HCTm (B) and between the ECVsyn and ECVm (E). HCTm strongly correlated with (HCTsyn –HCTm) (C) and the ECVm modestly correlated with (ECVsyn – ECVm) (F). (TIF 8817 kb)

Additional file 5: **Figure S3.** Comparison between the ECVm and ECVsyn in groups with the ECVm < 30.5 and > 30.5% for published model. In the group with the ECVm < 30.5%, the paired t-test demonstrated that the ECVsyn was larger than the ECVm in the derivation group (A) and validation group (C). In the group with the ECV > 30.5%, the paired t-test demonstrated that the ECVsyn did not differ with the ECVm in the derivation group (B) but smaller than the ECVm in validation group (D). (TIF 8151 kb)

Additional file 6: **Figure S4.** Comparison of the ECVsyn and ECVm among the healthy subjects and patients in the derivation group. The ECVm was larger in T2DMs patients than healthy subjects (A); however, the ECVsyn did not differ (A).Compared with the healthy subjects, the HCM patients had a higher ECVsyn and ECVm of the mid-ventricle (B) and interventricular septum (D). The ECVm and ECVsyn of the free wall in the HCM patients did not differ with those in the healthy subjects (C). (TIF 8317 kb)

Additional file 7: **Figure S5.** Comparison of the ECVsyn and ECVm among the controls and patients in the validation group. The ECVm was larger in T2DMs patients than healthy subjects (A); however, the ECVsyn did not differ (A). Compared with the healthy subjects, the HCM patients had a higher ECVsyn and ECVm of the mid-ventricle (B) and interventricular septum (D). The ECVm of the free wall in the HCM patients was larger than in the healthy subjects, but the ECVsyn was equivalent (C). (TIF 8326 kb)

## Abbreviations

ARVC: Arrhythmogenic right ventricular cardiomyopathy
BSA: Body surface area
bSSFP: Balanced steady state free precession
CI: Cardiac index
CMR: Cardiovascular magnetic resonance
DCM: Dilated cardiomyopathy
ECG: Electrocardiogram
ECV: Extracellular volume fraction
ECVm: Measured extracellular volume fraction
ECVsyn: Synthetic extracellular volume fraction
EDVi: End-diastolic volume index
EF: Ejection fraction
ESVi: End-systolic volume index
FOV: Field of view
HCM: Hypertrophic cardiomyopathy
HCT: Hematocrit
HCTm: Measured hematocrit
HCTsyn: Synthetic hematocrit
ICCs: Intra-class correlation coefficients
LGE: Late gadolinium enhancement
LV: Left ventricle/left ventricular
LVMi: Left ventricular mass index
LVNC: Left ventricular noncompaction
MOCO: Motion correction
MOLLI: Modified Look-Locker Inversion Recovery
PSIR: Phase sensitive inversion recovery
ROIs: Regions of interest
SVi: Stroke volume index
T2DM: Type 2 diabetes mellitus
