# Supplementary figures and images for: Extracellular volume fraction measurements derived from the longitudinal relaxation of blood-based synthetic hematocrit may lead to clinical errors in 3 T cardiovascular magnetic resonance
Source: J Cardiovasc Magn Reson. 2018 Aug 9;20:56. doi: 10.1186/s12968-018-0475-6 (PMC6083590; doi:10.1186/s12968-018-0475-6)

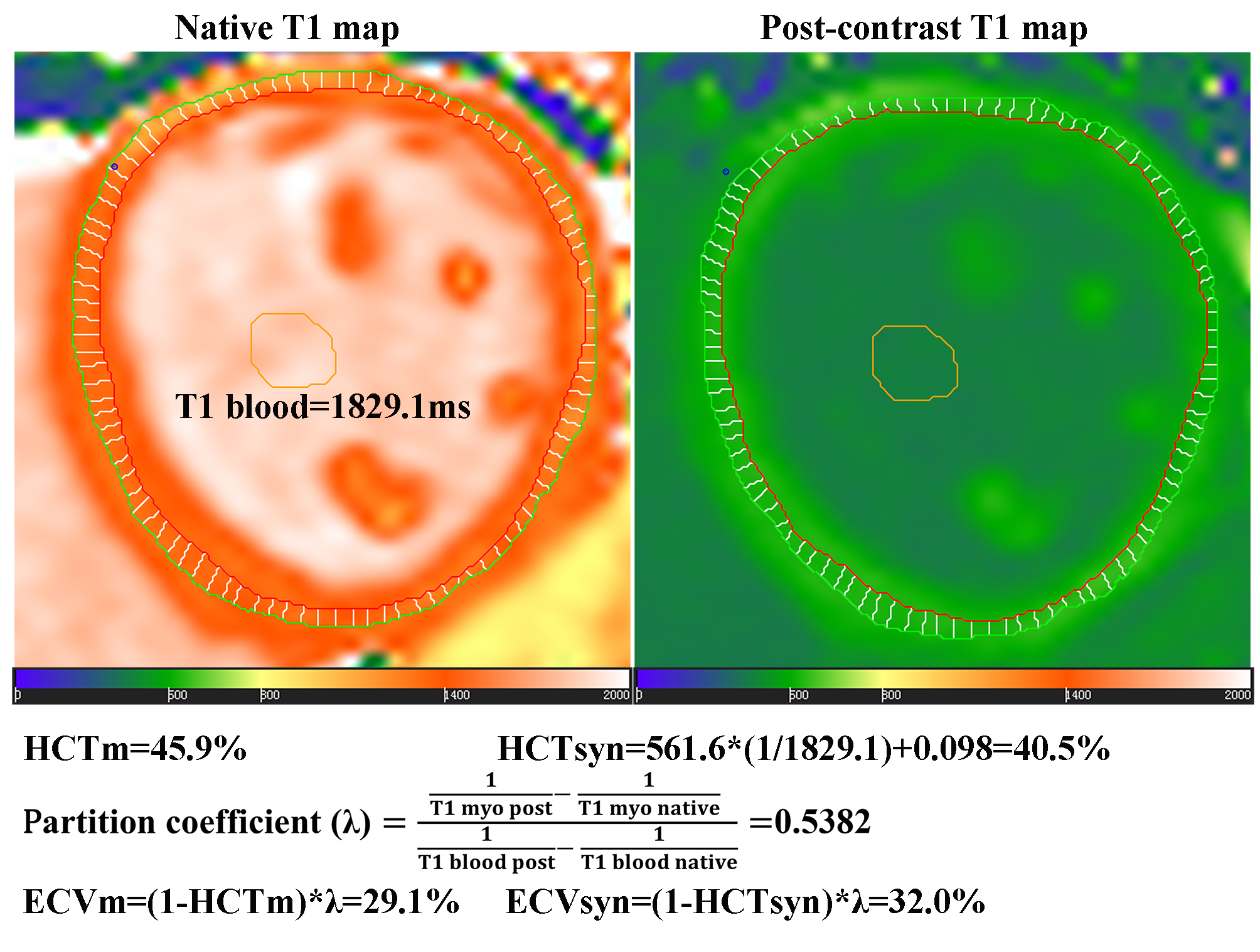

Supplement: Supplementary file 1 — Figure S7. Diagram of synthetic HCT and ECV analysis of the left ventricular myocardium in a participant. (TIF 5299 kb) [file 12968_2018_475_MOESM1_ESM.tif]

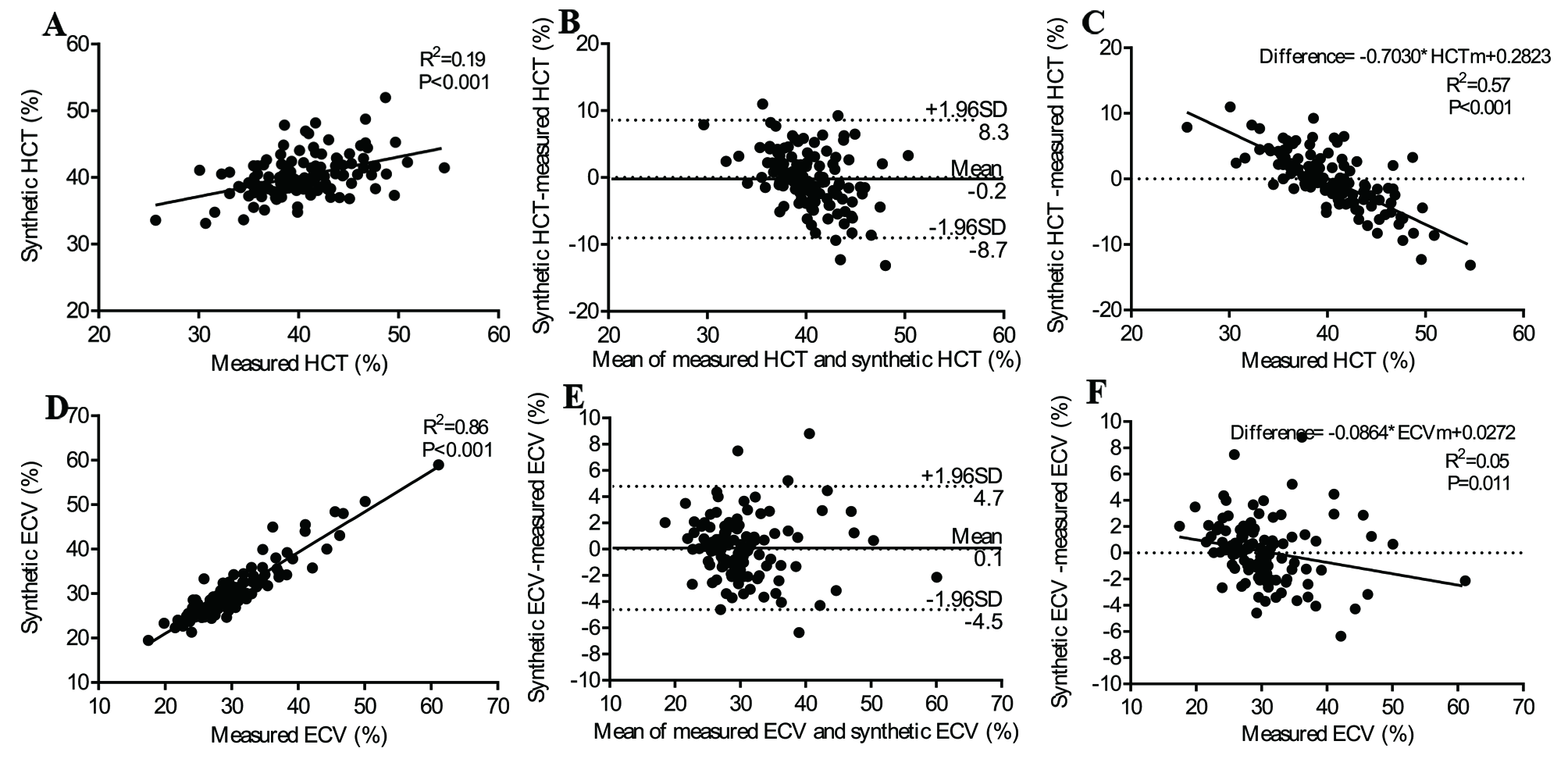

Supplement: Supplementary file 3 — Figure S1. Correlation among the HCTm, HCTsyn, ECVm, ECVsyn in the derivation group for published model. There was modest correlation between the HCTsyn and HCTm (A) and strong correlation between the ECVsyn and ECVm (D). Bland-Altman analysis indicated minimal bias between the HCTsyn and HCTm (B) and between the ECVsyn and ECVm (E). HCTm strongly correlated with (HCTsyn –HCTm) (C) and the ECVm poorly correlated with (ECVsyn – ECVm) (F). (TIF 8826 kb) [file 12968_2018_475_MOESM3_ESM.tif]

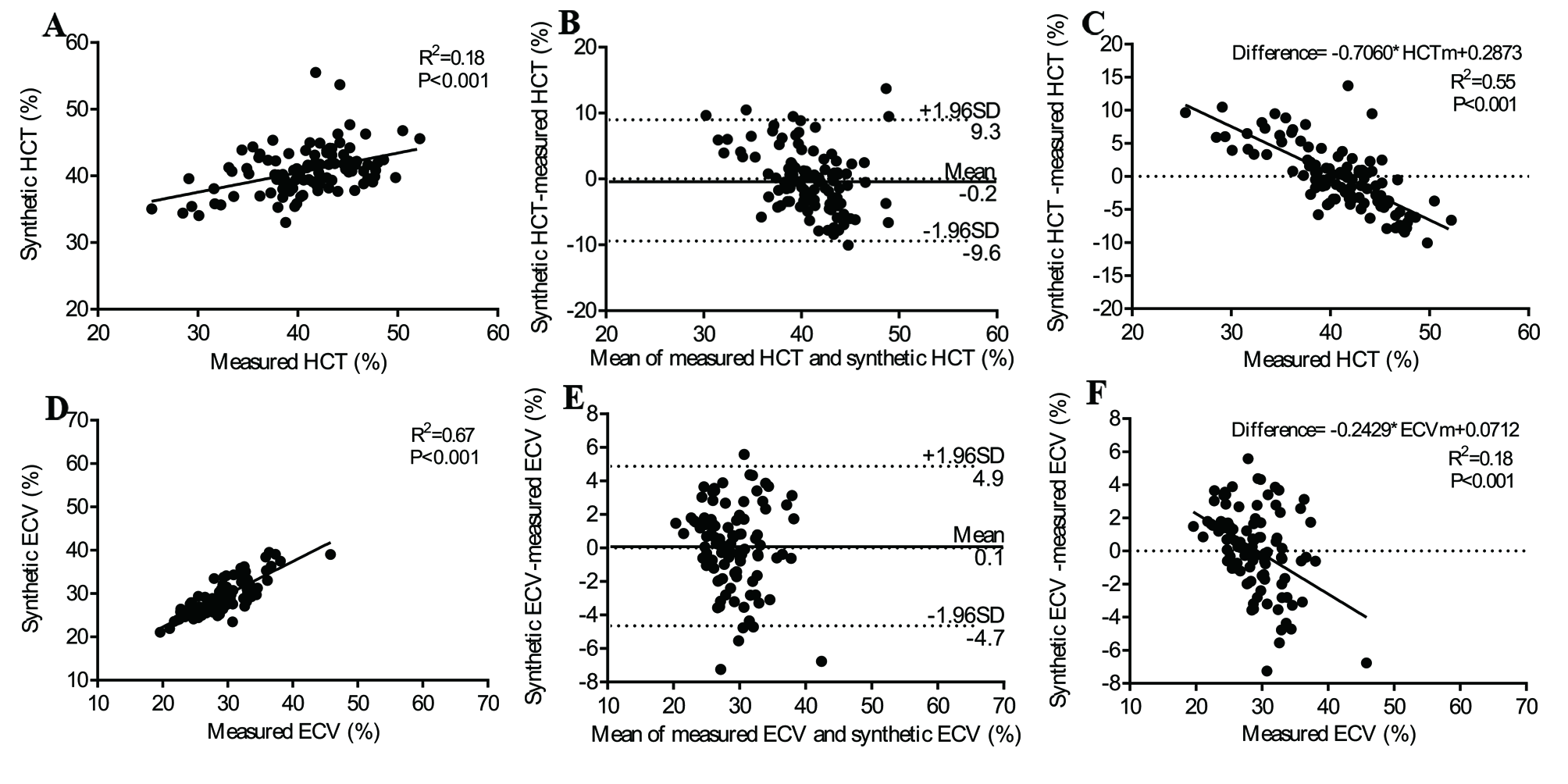

Supplement: Supplementary file 4 — Figure S2. Correlation among the HCTm, HCTsyn, ECVm, ECVsyn in the validation group for published model. There was modest correlation between the HCTsyn and HCTm (A) and strong correlation between the ECVsyn and ECVm (D). Bland-Altman analysis indicated minimal bias between the HCTsyn and HCTm (B) and between the ECVsyn and ECVm (E). HCTm strongly correlated with (HCTsyn –HCTm) (C) and the ECVm modestly correlated with (ECVsyn – ECVm) (F). (TIF 8817 kb) [file 12968_2018_475_MOESM4_ESM.tif]

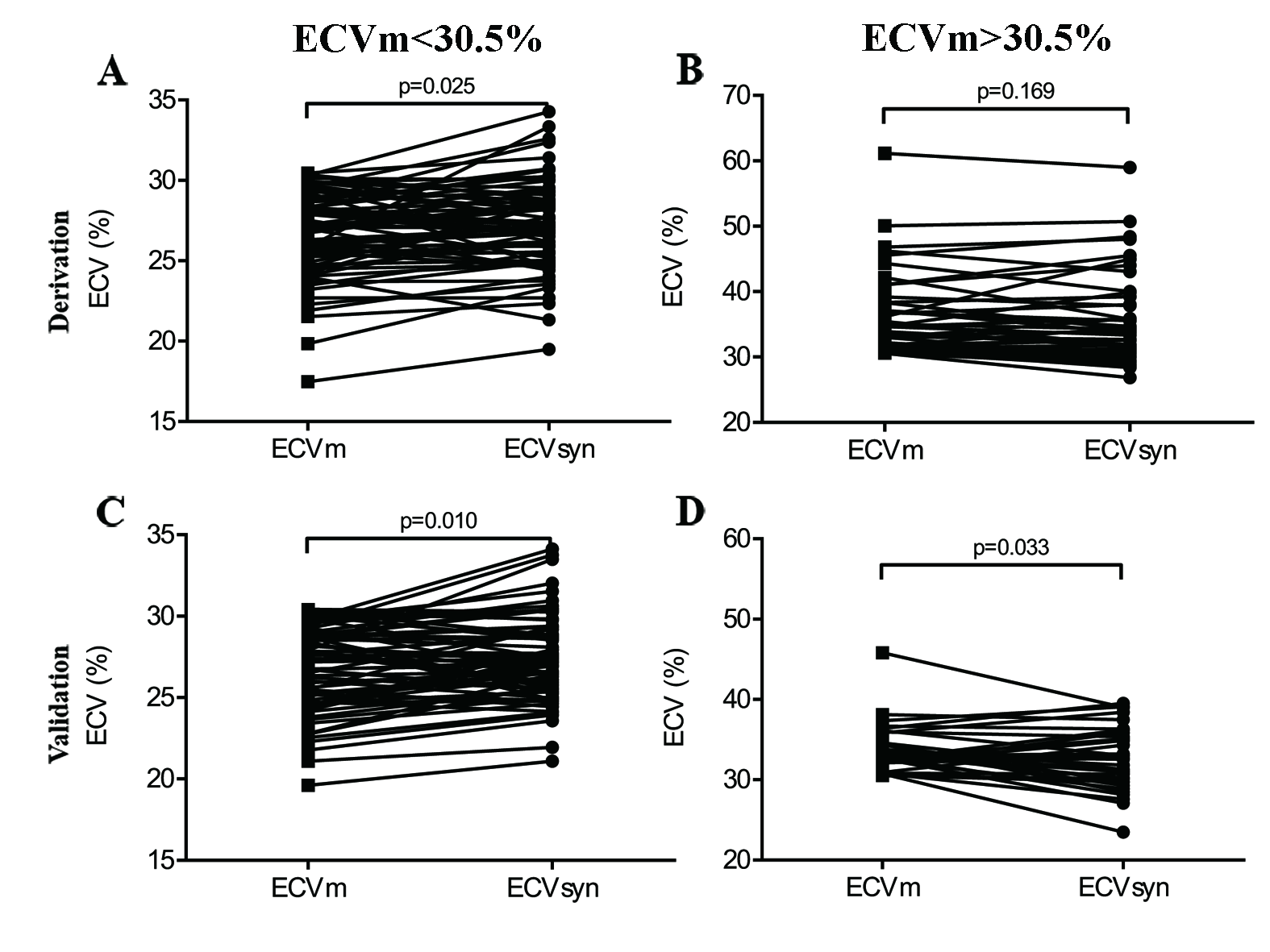

Supplement: Supplementary file 5 — Figure S3. Comparison between the ECVm and ECVsyn in groups with the ECVm < 30.5 and > 30.5% for published model. In the group with the ECVm < 30.5%, the paired t-test demonstrated that the ECVsyn was larger than the ECVm in the derivation group (A) and validation group (C). In the group with the ECV > 30.5%, the paired t-test demonstrated that the ECVsyn did not differ with the ECVm in the derivation group (B) but smaller than the ECVm in validation group (D). (TIF 8151 kb) [file 12968_2018_475_MOESM5_ESM.tif]

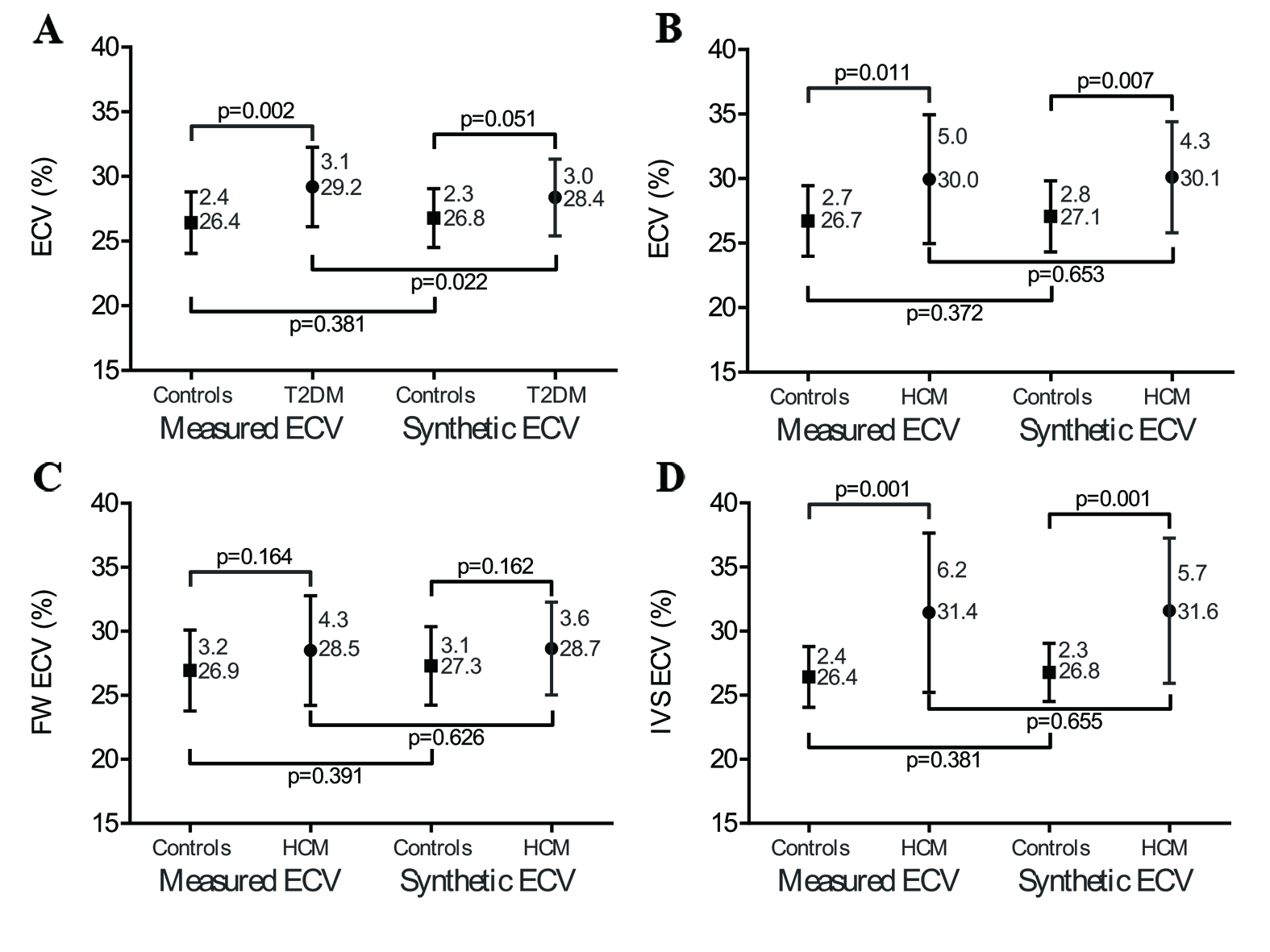

Supplement: Supplementary file 6 — Figure S4. Comparison of the ECVsyn and ECVm among the healthy subjects and patients in the derivation group. The ECVm was larger in T2DMs patients than healthy subjects (A); however, the ECVsyn did not differ (A).Compared with the healthy subjects, the HCM patients had a higher ECVsyn and ECVm of the mid-ventricle (B) and interventricular septum (D). The ECVm and ECVsyn of the free wall in the HCM patients did not differ with those in the healthy subjects (C). (TIF 8317 kb) [file 12968_2018_475_MOESM6_ESM.tif]

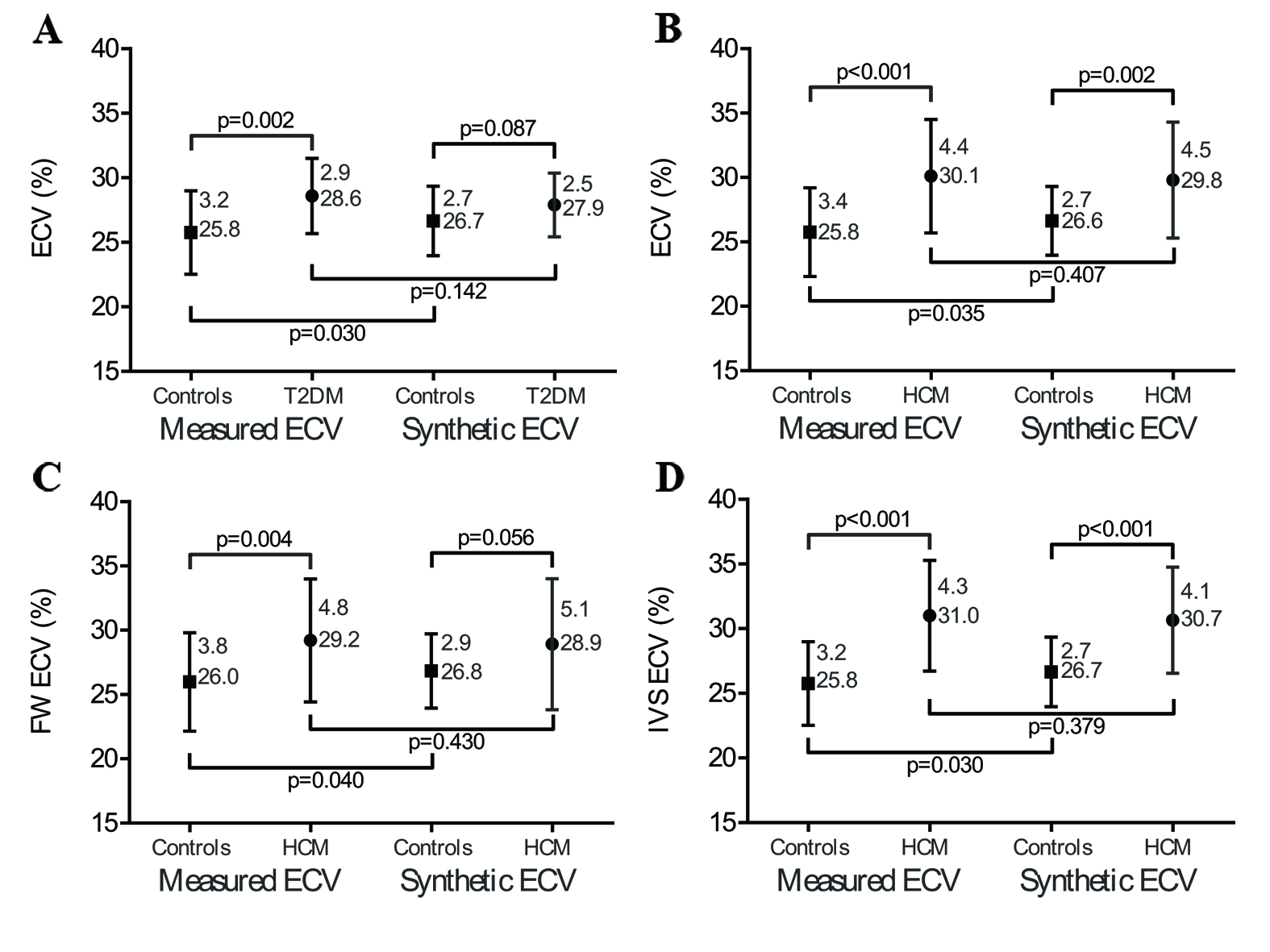

Supplement: Supplementary file 7 — Figure S5. Comparison of the ECVsyn and ECVm among the controls and patients in the validation group. The ECVm was larger in T2DMs patients than healthy subjects (A); however, the ECVsyn did not differ (A). Compared with the healthy subjects, the HCM patients had a higher ECVsyn and ECVm of the mid-ventricle (B) and interventricular septum (D). The ECVm of the free wall in the HCM patients was larger than in the healthy subjects, but the ECVsyn was equivalent (C). (TIF 8326 kb) [file 12968_2018_475_MOESM7_ESM.tif]
