# Supplementary material for: Extracellular volume fraction measurements derived from the longitudinal relaxation of blood-based synthetic hematocrit may lead to clinical errors in 3 T cardiovascular magnetic resonance
Source: J Cardiovasc Magn Reson. 2018 Aug 9;20:56. doi: 10.1186/s12968-018-0475-6 (PMC6083590; doi:10.1186/s12968-018-0475-6)
Supplement: Supplementary file 2 — Table S6. Mean difference and intraclass correlation coefficients (ICCs) for 16-segment extracellular volume fraction. (DOCX 13 kb) [file 12968_2018_475_MOESM2_ESM.docx]

| Supplementary Table 1. Mean difference and intraclass correlation coefficients (ICCs) for 16-segment extracellular volume fraction. | | | | | |
| --- | --- | --- | --- | --- | --- |
| AHA  Segments | Derivation | |  | Validation | |
|  | Mean difference (95%CI), 10^-4^ | ICC (95%CI) |  | Mean difference (95%CI) , 10^-4^ | ICC (95%CI) |
| 1 | -10(-51,31) | 0.932(0.899,0.955) |  | -7(-58,44) | 0.865(0.797,0.911) |
| 2 | -8(-50,34) | 0.930(0.896,0.953) |  | -1(-55,53) | 0.845(0.769,0.897) |
| 3 | -6(-46,34) | 0.931(0.897,0.954) |  | -2(-56,52) | 0.851(0.777,0.901) |
| 4 | -7(-48,34) | 0.941(0.912,0.961) |  | -8(-61,46) | 0.827(0.744,0.885) |
| 5 | -3(-43,37) | 0.945(0.918,0.963) |  | -7(-60,46) | 0.832(0.750,0.889) |
| 6 | -7(-45,32) | 0.909(0.865,0.939) |  | -4(-55,47) | 0.817(0.729,0.878) |
| 7 | -7(-47,32) | 0.931(0.897,0.954) |  | -4(-58,50) | 0.892(0.838,0.929) |
| 8 | -3(-45,38) | 0.921(0.882,0.947) |  | -7(-61,47) | 0.779(0.676,0.852) |
| 9 | -2(-43,38) | 0.931(0.897,0.954) |  | -6(-61,49) | 0.843(0.765,0.896) |
| 10 | -4(-43,34) | 0.921(0.882,0.947) |  | -9(-62,45) | 0.847(0.772,0.899) |
| 11 | -4(-43,35) | 0.921(0.883,0.947) |  | -14(-66,39) | 0.866(0.800,0.912) |
| 12 | -6(-44,33) | 0.885(0.830,0.922) |  | -8(-61,45) | 0.845(0.768,0.897) |
| 13 | -5(-47,37) | 0.936(0.905,0.958) |  | -5(-62,53) | 0.913(0.868,0.943) |
| 14 | -2(-44,39) | 0.939(0.909,0.960) |  | -9(-64,46) | 0.765(0.656,0.842) |
| 15 | 0(-40,40) | 0.939(0.909,0.959) |  | -12(-65,41) | 0.877(0.816,0.919) |
| 16 | -3(-44,38) | 0.932(0.898,0.954) |  | -9(-67,49) | 0.885(0.827,0.925) |
| 95%CI, 95% confidence Interval; ICC, intraclass correlation coefficient. | | | | | |
